# Nursing Graduates and Quality of Acute Hospital Care in 33 OECD Countries: Evidence From Generalized Linear Models and Data Envelopment Analysis

**DOI:** 10.1177/23779608211005217

**Published:** 2021-03-31

**Authors:** Arshia Amiri

**Affiliations:** 1 Department of Nursing Science, University of Turku, Turku, Finland; 2 School of Health and Social Studies, JAMK University of Applied Sciences, Jyväskylä, Finland

**Keywords:** acute myocardial infarction, hemorrhagic stroke, ischemic stroke, effectiveness, graduate nurses, staffing level

## Abstract

**Background:**

There is a lack of cross-national research to examine the role of new graduate nurses in improving the quality of nursing care and patient outcomes.

**Purpose:**

To measure the role and clinical effectiveness of new graduate nurses in improving the quality of acute hospital care in the members of Organisation for Economic Co-operation and Development (OECD).

**Methods:**

The total number of nursing graduates per 100,000 population and three OECD’s Health Care Quality Indicators (HCQI) in acute care including 30-day in-hospital and out-of-hospital mortality rates per 100 patients based on acute myocardial infarction (MORTAMIO), hemorrhagic stroke (MORTHSTO) and ischemic stroke (MORTISTO) were collected in 33 OECD countries. Four control variables including the number of medical graduates, practicing nurses and doctors densities per 1000 population (proxies for other health professions) and the total number of Computed Tomography scanners per one million population (proxy of medical technology level) were added in investigations. The statistical technique of Generalized Linear Models (GLM) and Data Envelopment Analysis (DEA) were used in data analysis.

**Results:**

Results of GLM confirm the existence of meaningful association between the density of nursing graduates and improving the quality of acute care i.e. a 1% rise in the number of nursing graduates in year 2015 reduced MORTAMIO, MORTHSTO and MORTISTO by 1.11%, 0.08% and 0.46%, respectively. According to the result of DEA, clinical effectiveness of new graduate nurses – i.e. reaching the higher clinical outcomes with the same staffing level – in reducing mortality rates in patients with life-threatening conditions were at highest level in Luxembourg, Finland, Japan, Italy, Norway, Sweden and Switzerland.

**Conclusions:**

Higher staffing level of new graduate nurses associates with better patient outcomes in acute care, although the clinical effectiveness of nursing graduates – associated with the level of education and practice – is the determinant factor of improving the quality of acute hospital care and patient survival rates in OECD.

## Introduction

Despite the noticeable rise in level of nurse staffing among the members of Organisation for Economic Co-operation and Development (OECD) from the average of 7.3 per 1000 inhabitants in 2000 to 9 per 1000 inhabitants in 2015, there are growing concerns for future nursing shortages in all OECD countries associated with demographic changes such as aging and retirement of a current generation of nurses who are at the highest level of labor efficiency (OECD, 2017b). For instance, the density of practicing nurses per inhabitants has dropped since 2000 in Slovak Republic, followed by Ireland, Israel and United Kingdom.

To reduce the disadvantages of current and future nursing shortage as well as to ensure proper care delivery, most of OECD countries tried to raise the number of graduate nurses and improve the efficiency and effectiveness of nursing education over the past decade (OECD, 2016). However, there are wide variations in efforts to train newly graduated nurses across developed countries according to the factors like a) distinction in the number and age of the current practicing nurses and the necessity of replacement, b) the future employment prospects of nursing in the process of health reformations as well as the capacity of nursing schools to admit higher number of nursing students, and c) the level of nursing education (OECD, 2017a).

**Figure 1.**
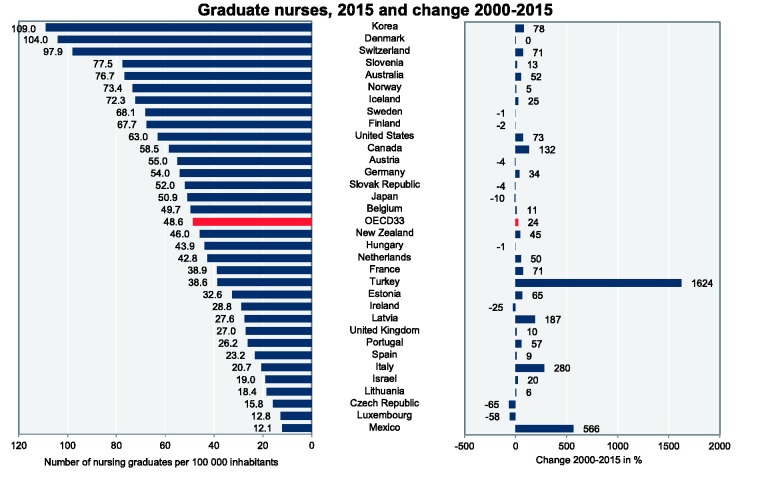
Number of Nursing Graduates per 100,000 Population, 2015 and Change 2000–2015. *Source*: OECD (2019a).

Since 2000, the number of graduate nurses has risen with different rates in many of OECD countries – strongly observed in Turkey, Mexico and Italy –, except in Czech Republic, Luxembourg, Ireland, Japan, Austria, Slovak Republic, Finland, Hungary and Sweden – see Figure 1. France has shown a rise in the number of graduate nurses (by 71%) due to substantial expansion in nursing education programs by the French Ministry of Health since 1999, driven by concerns about the predicted retirement of many practicing nurses as well as a projected policy to reduce nurse-staffing level resulting from reduction in working hours (OECD, 2017a). In Germany, the number of graduates from nursing programs increased by 34% in the last decade through the development of registered nurse education programs from traditional vocational nursing schools to several universities (Cassier-Woidasky, 2013). Although there was a reduction in the trend of graduate nurses in Japan, Finland and Norway during 2005-2010, these countries showed a moderate rise in the number of new graduate nurses in the last few years (OECD, 2017a).

In response to nursing shortages and to improve the quality of healthcare delivery in health facilities, it is critical for health policymakers along with researchers to work toward finding more efficient nursing education systems with the aim of expanding the level of graduate nurses and advancing the quality of training new nurses. To do this, the first step is to determine and compare the effect of graduate nurses on improving patient outcomes, which can be considered as a proxy for quality of nursing education among OECD countries.

To our knowledge, the role nursing and nursing-related services in improving patient outcomes and quality of care have been confirmed at a national level by empirical studies like Aiken et al. (2014), Wiig et al. (2014), Aiken et al. (2018), Coster et al. (2018), Amiri and Solankallio-Vahteri (2019a, 2019b), Freeling et al. (2020), Amiri et al. (2019, 2020) and Amiri (2020). However, there is a lack of cross-national research to examine and measure the role of new graduate nurses in improving the quality of nursing care and clinical outcomes in OECD.

This study aimed to examine the impact of increasing staffing levels, secondary to new graduate nurse employment, on the quality of acute hospital care services. The data analysis of the following study has two parts. Firstly, the statistical technique of generalized linear models (GLM) is conduced to investigate the possible association between the staffing level of new gradate nurses and OECD’s Health Care Quality Indicators (HCQI) i.e. reducing 30-day in-hospital and out-of-hospital deaths per 100 patients based on acute myocardial infarction (AMI), hemorrhagic and ischemic strokes in 33 OECD countries. Secondly, Data Envelopment Analysis (DEA) is applied to quantify the clinical effectiveness of nursing graduates – defined by reaching the higher clinical outcomes with the same staffing level – in improving the quality of acute care in OECD.

## Data Description

**Figure 2.**
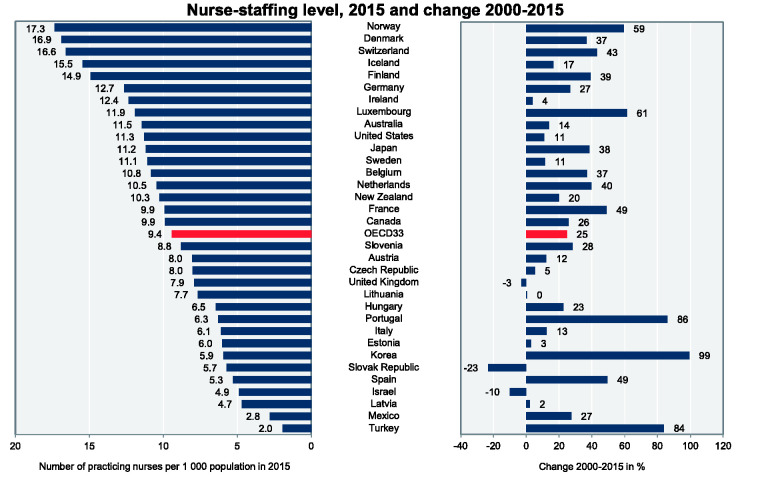
Number of Practicing Nurses per 1000 Population, 2015 and Change 2000–2015. *Source*: OECD (2019b).

Observations of total number of nursing graduates per 100,000 population together with the number of practicing professional nurses’ density per 1000 population, including general care nurses, specialist nurses, clinical nurses, district nurses, nurse anesthetists, nurse educators, nurse practitioners and public health nurses, as the indexes of staffing levels of new graduate nurses and practicing nurses – see Amiri and Solankallio-Vahteri (2020) –, were collected from OECD (2019c, 2019d) for 33 OECD countries in 2015. Figures 1 and 2 depict the column charts of staffing levels of graduate nurses and practicing nurses in 2015 and changes since 2000 in OECD countries.

**Figure 3.**
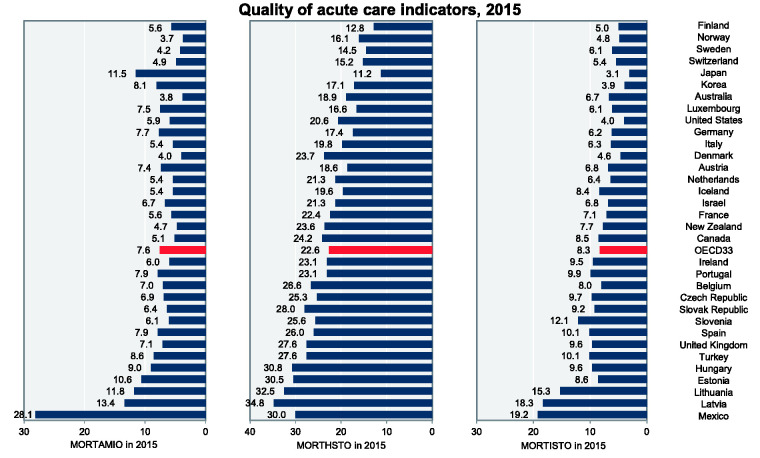
Number of 30-day In-Hospital and Out-of-Hospital Mortality per 100 Patients Based on AMI (MORTAMIO), Hemorrhagic Stroke (MORTHSTO) and Ischemic Stroke (MORTISTO) in 2015. *Source*: OECD Health Statistics (2019a).

OECD’s HCQI are the number of 30-day – after the first admission to hospital – in-hospital and out-of-hospital deaths per 100 patients in 2015 based on acute myocardial infarction (AMI) mortality (MORTAMIO) with diagnostic codes ICD-9 410 or ICD-10 I21, I22, hemorrhagic stroke mortality (MORTHSTO) with diagnostic codes ICD-9 430-432 or ICD-10 I60-I62 along with ischemic stroke mortality (MORTISTO) with diagnostic codes ICD-9 433, 434, and 436 or ICD10 I63-I64. OECD’s HCQI covered 45 years old and over admitted patients and verified age-sex standardized and collected from OECD Health Statistics (2019a) to measure the quality of acute hospital care according to national hospital inpatient administration. Figure 3 depicts the variation of OECD’s HCQI in 2015 among countries.

In addition, the observations of medical graduates per 100,000 population collected from OECD (2019b), total number of practicing doctors per 1000 population gathered from OECD (2019a) as proxies for other health workforces and the total number of Computed Tomography scanners per million population as the proxy of medical technology level available at OECD Health Statistics (2019b) were applied in our analysis as control variables.

## Generalized Linear Models

Nelder and Wedderburn (1972) proposed a generalization version of linear regression models (GLM) with the advantage of regressing non-linear forms of systematic ingredients, i.e. exponential, logistic and probit regressors as well as Poisson models with the ability to regress variables with error distribution models or non-normal stochastic distributions. Additionally, the GLM furnished a modified statistical structure to generalize linear regressors by eliminating the traditional assumptions and limitations according to the distributions of the variable participated in statistical analysis and opened the way to simulate variables with the possible correlation between the variance of each coefficient and its estimated values – for more details about GLM see McCullagh and Nelder (1989) and Hardin and Hilbe (2007).

This study applies GLM with the flexibility of regressing nonlinear models – instead of classical cross-sectional methods used in nursing – to investigate statistically significant association between the staffing level of nursing graduates and OECD’s HCQI. Results of GLM are available in Table 1 and verify that the coefficients of graduate nurses according to LR statistics were statistically meaningful at conventional levels i.e. there was a meaningful association between the staffing level of new graduate nurses and reducing 30-day mortality rates associated with AMI, hemorrhagic and ischemic strokes in OECD. In other words, a 1% rise in the staffing level of nursing graduates may reduce MORTAMIO, MORTHSTO and MORTISTO by 1.11%, 0.08% and 0.46%, respectively. In addition, the coefficients of other health professions including practicing nurses, doctors were statistically and theoretically meaningful except new medical graduates.

**Table 1.** Results of GLM Analysis (33 OECD Countries, 2015).

Variable	Coefficient	Std. Error	z-Statistic	Prob.	LR statistic	LR prob.
Dependent variable: MORTAMIO						
Constant	3.406657	0.017917	190.1407	0.0000	24.37799	0.0002
Graduate Nurses	−0.01110	0.000258	−42.9511	0.0000		
Nurse-staffing level	−0.06825	0.000694	−98.3623	0.0000		
Physician-staffing level	−0.21232	0.004232	−50.1667	0.0000		
Medical graduates	0.013566	0.000360	37.69910	0.0000		
Medical technology indicator	0.007560	0.000106	71.53920	0.0000		
Dependent variable: MORTHSTO						
Constant	3.345514	0.000335	9982.522	0.0000	43.53283	0.0000
Graduate Nurses	−0.00075	2.33E−06	−321.511	0.0000		
Nurse-staffing level	−0.03455	2.24E−05	−1540.56	0.0000		
Physician-staffing level	−0.02634	0.000103	−256.148	0.0000		
Medical graduates	0.024285	1.54E−05	1579.055	0.0000		
Medical technology indicator	−0.00363	4.98E−06	−728.687	0.0000		
Dependent variable: MORTISTO						
Constant	2.488599	0.004766	522.1168	0.0000	47.19861	0.0000
Graduate Nurses	−0.00456	4.97E−05	−91.7621	0.0000		
Nurse-staffing level	−0.06781	0.000535	−126.819	0.0000		
Physician-staffing level	−0.04941	0.001161	−42.5497	0.0000		
Medical graduates	0.049431	0.000325	151.8801	0.0000		
Medical technology indicator	−0.00171	8.08E−05	−21.1609	0.0000		

Notes: GLM were based on Newton-Raphson method with Marquardt steps including 33 observations for each regression. Family was selected normal and link was log. Dispersion of LR statistics and probabilities calculated based on Pearson Chi-Square criterions. Coefficient covariance estimated by Newey-West HAC method using Hessian (Bartlett kernel, Newey-West fixed bandwidth = 4.00).

## Data Envelopment Analysis (DEA)

DEA commonly used to assess the efficiency of decision-making units (DMUs). It is a nonparametric empirical approach to investigate the performance of DMUs participated in the statistical analysis due to explain the variation of endogenous variables. DEA is a two-stage data analysis method; firstly, DEA calculates the *best-practice frontier* – i.e. hypothetical frontier line – with identifying the extreme amounts of output or endogenous variable that is possible to achieve by the minimum amounts of inputs or exogenous variables. Secondly, by estimating frontier function the efficiency rates of every DMU can be investigated according to the hypothesis that if a DMU reached a certain level of output with employing the certain level of inputs, then the other DMUs should be capable to do the same.

Here, DEA is applied to measure the clinical effectiveness of nursing graduates which defined by reaching the higher clinical outcomes with the same staffing level. Technically, DEA calculates the maximum effect of graduate nurses (exogenous variable) on improving the quality of acute care indicators (dependent variables) to find the countries that reached the lowest mortality rates in acute care with the same level of staffing and subsequently it measures the efficiency rates of each OECD country (DMU).

**Figure 4.**
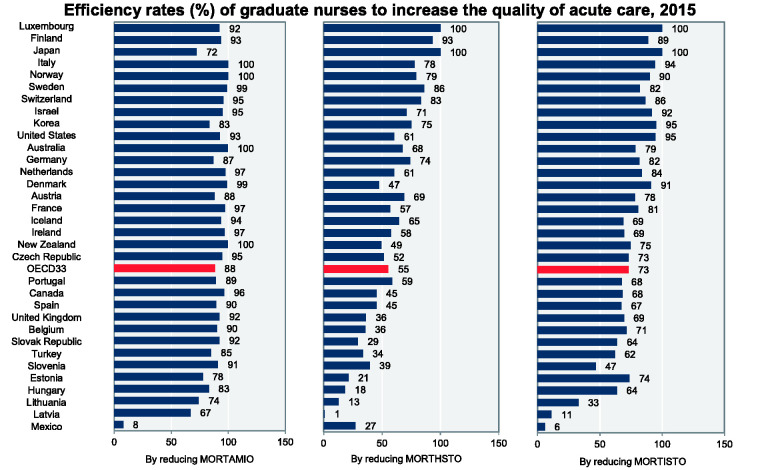
Efficiency Rates (%) of New Graduate Nurses in Reducing 30-Day In-Hospital and Out-of-Hospital Mortality per 100 Patients Based on AMI (MORTAMIO), Hemorrhagic Stroke (MORTHSTO) and Ischemic Stroke (MORTISTO), as the Results of DEA in 33 OECD Countries, 2015.

Results of DEA are available in Table 2 and Figure 4 and argue that the highest clinical efficiency of graduate nurses in reducing MORTAMIO were calculated in Italy and Norway (100%), followed by Australia (99.62%), New Zealand (99.55%), Denmark (98.86%) and Sweden (98.75%). For the rest of OECD countries, the efficiency rates of new graduate nurses in decreasing AMI-based mortality rate were in the range between 97.19% in Netherlands and 66.88% in Latvia, except Mexico with lowest efficiency rate of only 7.81%.

**Table 2.** Results of DEA (33 OECD Countries, 2015).

Efficiency rates (%) of graduate nurses in reducing MORTAMIO, MORTHSTO and MORTISTO.				
Country	MORTAMIO	MORTHSTO	MORTISTO	Average
Australia	99.619	67.647	78.698	81.988
Austria	87.920	68.907	78.106	78.311
Belgium	90.075	35.556	71.418	65.683
Canada	96.441	45.378	68.047	69.955
Czech Republic	94.510	51.549	72.874	72.978
Denmark	98.859	47.478	91.124	79.154
Estonia	77.650	21.223	73.746	57.540
Finland	93.438	93.277	88.757	91.824
France	96.880	57.037	80.871	78.263
Germany	86.856	73.949	81.656	80.821
Hungary	82.850	18.421	63.628	54.966
Iceland	93.672	64.705	68.639	75.672
Ireland	96.542	57.612	69.287	74.480
Israel	94.927	71.086	91.751	85.921
Italy	100.000	77.900	94.354	90.752
Japan	72.338	100.000	100.000	90.779
Korea	83.269	75.210	95.266	84.582
Latvia	66.878	0.9762	11.287	26.380
Lithuania	74.205	13.027	32.773	40.002
Luxembourg	92.418	100.000	100.000	97.480
Mexico	7.8114	27.327	5.7783	13.639
Netherlands	97.190	60.505	83.653	80.449
New Zealand	99.550	49.358	74.502	74.470
Norway	100.000	79.411	89.940	89.784
Portugal	89.192	58.630	67.535	71.786
Slovak Republic	92.155	29.411	63.905	61.824
Slovenia	90.874	39.495	46.745	59.038
Spain	89.546	45.305	67.274	67.375
Sweden	98.749	86.134	82.248	89.044
Switzerland	95.437	83.193	86.390	88.340
Turkey	84.996	33.552	62.141	60.229
United Kingdom	92.328	36.264	69.260	65.951
United States	92.818	60.504	94.674	82.665
OECD33	88.182	55.456	72.919	72.186

Notes: Frontier functions were calculated based on variable return to scale (VRS) method.

Luxemburg and Japan with 100%, followed by Finland with 93.28%, Sweden with 86.13%, Switzerland with 83.19% and Norway with 79.41% had the most efficient nursing care provided by new graduate nurses in reducing MORTHSTO. By contrast, the lowest efficiency rates of new nursing graduates were simulated for Slovak Republic, Mexico, Estonia, Hungary, Lithuania and Latvia with less than 30%.

In MORTISTO, again Luxemburg and Japan had the most efficient nursing care in declining 30-day ischemic stroke-based mortality rates by new graduate nurses, followed by Korea (95.27%), United States (94.67%), Italy (94.35) and Israel (91.75%). By contrast, the lowest amounts of graduate nurses’ efficiency rate were calculated in Slovenia with 46.74%, Lithuania with 32.77%, Latvia 11.29% and Mexico with 5.78%.

For all OECD countries which the data were available, the average amounts of efficiency of nursing graduates in reducing MORTAMIO (88.18%) and MORTISTO (72.92%) were slightly more than MORTHSTO (55.45%). Overall, the average efficiency rates of graduate nurses in reducing mortality rates in patients with acute and life-threatening conditions were at highest level in Luxembourg (97.48%), Finland (91.82%), Japan (90.78%), Italy (90.75%), Norway (89.78%), Sweden (89.04%) and Switzerland (88.34%). By contrast, the efficiency rates of graduate nurses were at lowest level among developed countries in Estonia with 57.54%, Hungary with 54.96%, Lithuania with 40.00%, Latvia with 26.38% and Mexico with 13.64%.

## Discussion

There has been much interest in analyzing the association between the staffing level of new graduate nurses and improving the quality of acute hospital care. Although there is no doubt in the significant impacts of practicing nurses in improving the quality of acute care, the role of new graduate nurses on improving the quality of care, reducing safety failure and patient outcomes has not been researched in cross-national level. To our knowledge, most of studies focused on the factors that influence nursing education and new graduate nurses’ practical skills, including but not limited to Guo et al. (2018), Jones et al. (2020), Jamieson et al. (2020), Mansour et al. (2020), Lockhart et al. (2020), Christensen et al. (2020), Mokel and Canty (2020) etc.

This study started a new attempt in nursing science to evaluate the effect of nursing graduates due to their level of staffing – i.e. as a proxy for the effectiveness of nursing education –, on improving the quality of OECD’s HCQI using cross-national statistics of 33 countries. The results of GLM argued that there were significant associations between the staffing level of new graduate nurses and reducing 30-day in-hospital and out-of-hospital deaths per 100 patients based on acute myocardial infarction (AMI), hemorrhagic and ischemic strokes in OECD. Furthermore, results of DEA verified that the effectiveness of graduate nurses in reducing mortality rates in patients with life-threatening conditions – quantified by reaching the higher clinical outcomes with the same staffing level – were estimated at highest level in Luxembourg, Finland, Japan, Italy, Norway, Sweden and Switzerland among OECD countries.

In all, the findings of this study prove that although the higher proportion of new graduate nurses is associated with lower mortality rates and better clinical outcomes in acute care, the efficiency of nursing graduates – which is associated with the quality of nursing education – plays a key role in maximizing nursing outcomes, decreasing the risk of complication, mortality and clinical failures i.e. better educated nurses can work more efficiently. The recommendation of this study for health policymakers, health educators and health professionals is to follow the nursing education models and practicing systems of Luxembourg, Finland, Japan, Italy, Norway, Sweden and Switzerland to optimize the quality of nursing care in both national and global levels. Moreover, our results alert health policymakers to consider the burden of nursing shortage in health care systems of OECD countries resulting from increasing adverse clinical outcomes and complications. Hence, there is a need of educational and fiscal policies with the aim of rising the number new nursing graduate as well as quality of training new nurses.

Due to the lack of available data, the limitation of this study was the lack of considering the effect of related factors like the proportion of medical/surgical nurses, the characteristics of nursing education in different OECD countries, clinical practices etc. in the data analysis. The principal direction of future research would be to study what agents stimulate the clinical efficiency of new graduate nurses along with monitoring and analyzing the trends of nursing graduates across OECD countries to prevent nursing shortage. According to the lack of cross-national research in nursing science especially in educational topics, it is recommended by global organizations like OECD and World Health Organization (WHO) to collaborate with researchers to support countries in collecting and analyzing national and international clinical observations (Amiri et al., 2012; Amiri & Linden, 2016; Amiri & Ventelou, 2012).

## Conclusion

The higher staffing level of nursing graduates is associated with higher quality of acute care in OECD countries, although the clinical effectiveness of nursing graduates – associated with the quality of nursing education and practice – is the key factor of improving nursing outcomes in the care of patients with life-threatening conditions.
